# Association between care complexity individual factors and older inpatients with COVID-19: a cross-sectional study

**DOI:** 10.3389/fragi.2025.1524849

**Published:** 2025-08-05

**Authors:** Jordi Adamuz, Julia González-Vaca, Maribel González-Samartino, María-Magdalena López-Jiménez, Andrea Urbina, Oliver Polushkina-Merchanskaya, Sergio Alonso-Fernández, Silvia Esteban-Sepúlveda, Sergio Barrientos-Trigo, Maria-Eulàlia Juvé-Udina

**Affiliations:** ^1^ Nursing Knowledge Management and Information Systems Department, Bellvitge University Hospital, L’Hospitalet de Llobregat, Barcelona, Spain; ^2^ Faculty of Nursing, University of Barcelona, L’Hospitalet de Llobregat, Barcelona, Spain; ^3^ Bellvitge Institute of Biomedical Research (IDIBELL), L’Hospitalet de Llobregat, Barcelona, Spain; ^4^ Nursing Department, University of Seville, Seville, Spain; ^5^ Nursing Management Team, Catalan Institute of Health, Barcelona, Spain

**Keywords:** care complexity, patient assessment, older adults, hospitalization, COVID-19

## Abstract

**Background:**

Many elderly people required hospitalization during the pandemic period, but broader care complexity factors have not been studied in this population. This study aimed to identify the care complexity factors according to age in older people hospitalized with COVID-19.

**Methods:**

A multicenter cross-sectional study was conducted from 1 March 2020 to 31 March 2022 at eight public hospitals in Spain. All older patients hospitalized with COVID-19 were classified in the following groups: young-old (65–74 years), middle-old (75–84 years), and oldest-old (≥85 years). The main variable was care complexity individual factors (CCIFs), which included 27 CCIFs classified in four domains: comorbidity/complications, psycho-emotional, mental-cognitive, and sociocultural. Multinomial logistic regressions were performed to identify the association of each CCIFs with age group.

**Results:**

A total of 5,658 admissions were included. Of these, 46.3% were young-old (65–74 years), 34.8% middle-old (75–84 years) and 18.8% oldest-old (≥85 years). The analysis shows that middle-old (75–84 years) patients were associated with chronic disease, position impairment, urinary or fecal incontinence, anatomical and functional disorders, vascular fragility, involuntary movements, fear or anxiety and mental status impairments. Extreme weight, communication disorders, aggressive behavior, agitation and perception reality disorders were additional factors associated with the oldest-old (≥85 years) inpatients with COVID-19. The median number of CCIFs was higher in the oldest-old than in the other age groups (four in young-old [65–74 years]; six in middle-old [75–84 years]; seven in oldest-old [≥85 years] [OR:2.9; 95%CI:2.8–3.1; p < 0.001]).

**Conclusion:**

The oldest groups of patients (≥75 years) admitted with COVID-19 had more CCIFs than the young-old group. CCIFs should be included in patient assessment in order to identify care needs in older hospitalized patients.

## Highlights

Many older adults required hospitalization during the pandemic period, however CCIFs have not been studied in this population.

We found that the oldest groups of patients (≥75 years) admitted with COVID-19 had more CCIFs than the young-old group.

This study strengthens the argument for considering broader patient assessment to identify care complexity needs.

## Introduction

The COVID-19 pandemic disproportionately affected individuals over 65 years old. According to data from Spain, more than 60% of COVID-19 patients hospitalized during the first months of the pandemic were aged over 65 years, and this demographic group accounted for approximately 85% of deaths from the disease (Instituto Nacional de Estadística. Instituto Nacional de Estadística INE, 2024).

Prior studies focusing on older people hospitalized with COVID-19 focused on identified risk factors and frailty indicators associated with unfavorable outcomes (Wiemers et al., 2024; Wei et al., 2020; Szklarzewska et al., 2023; Alsahab et al., 2021; Smits et al., 2022) however, broader care complexity factors have not been studied in this population. Understanding care complexity individual factors (CCIFs) is crucial for developing more effective and personalized management strategies for older adults with COVID-19. CCIFs are a set of patient characteristics related to different health dimensions that may complicate care delivery and contribute to adverse events (Juvé-Udina et al., 2010). Previous studies have shown the association of CCIFs with unfavorable outcomes in hospitalized patients in general and in those admitted for COVID-19 (Jiménez-Martínez et al., 2024; Adamuz et al., 2018; Adamuz et al., 2021). Therefore, prior identification of CCIFs could help to identify patients with a higher risk of complications (Adamuz et al., 2020). The use of assessment tools considering these factors could improve care planning accuracy and optimize resource allocation. This approach could inform public health policies aimed at strengthening the health system’s capacity to respond to future health emergencies and the needs of the aging population (De Foo et al., 2023). However, few studies have provided data about CCIFs in the vulnerable elderly population hospitalized with COVID-19.

The aim of this study was to identify CCIFs according to the age of older adults hospitalized with COVID-19.

## Methods

### Study population

A multicenter cross-sectional study was conducted from 1 March 2020 to 31 March 2022 at eight public health hospitals in Spain: three high-tech hospital (tertiary metropolitan facilities), three urban university centers and two community hospitals. All patients with a medical diagnosis of COVID-19 infection admitted to a ward or step-down unit from 1 March 2020 to 31 March 2022 with a completed hospital minimum data set report were included. Patients directly admitted and discharged from intensive care units (ICU) were excluded.

This study was evaluated and approved by the institutional review board. Ethical and data protection protocols related to anonymity and data confidentiality (access to records, data encryption and archiving of information) were complied with throughout the study.

### Data source

All data were collected from the electronic health record system, the hospital minimum data set and the clinical data warehouse. A unique identification number was used to link the data sets from these sources.

### Variables

Consistent with the literature, older adults were categorized as young-old (65–74 years), middle-old (75–84 years), and oldest-old (≥85 years) (Neugarten, 1974; Johfre and Saperstein, 2023). The main variable was care complexity individual factors (CCIFs) (Juvé-Udina et al., 2010). CCIFs are a group of patient characteristics related to different health dimensions that may complicate care delivery and contribute to adverse outcomes. They are classified into four domains: (i) comorbidity/complications, (ii) psycho-emotional, (iii) mental-cognitive sociocultural, and iv) sociocultural. Patients were considered to have CCIFs if they presented at least one related defined characteristic according to previous inquiries. CCIFs were collected from the nursing assessment e-charts as structured data based on the Architecture, Terminology, Interface, Knowledge (ATIC) terminology (Juvé-Udina and Adamuz, 2023; Juvé-Udina, 2013). In this study we included the following CCIFs. The comorbidity/complications domain contained: (i) major chronic disease, (ii) hemodynamic instability (intensive control of vital signs or state of shock), (iii) high risk of haemorrhage (coagulation disorders, thrombocytopenia, anticoagulant therapy), (iv) communication disorders (aphasia, dysphasia, dysarthria, laryngectomy, tracheostomy), (v) urinary or faecal incontinence, (vi) vascular fragility (capillary fragility, tortuous veins), (vii) position impairment, (viii) involuntary movements (continuous involuntary movements), (ix) extreme weight (low weight, obesity), (x) dehydration (skin turgor), (xi) oedema, (xii) uncontrolled pain (verbal numerical rating scale above three points), (xiii) transmissible infections (isolation measures), (xiv) immunosuppression and (xv) anatomical and functional disorders (amputation, deformities, joint stiffness). The psycho-emotional domain comprised: (i) aggressive behaviour, (ii) fear/anxiety and (iii) impaired adaptation (disruptive behaviour, hopelessness or surrender). The mental-cognitive domain included: (i) agitation, (ii) mental status impairments (confusion, disorientation, stupor, transient loss of consciousness), (iii) impaired cognitive functions (intellectual disability, amnesia) and (iv) perception of reality disorders (delirium, hallucinations, disconnection from reality). Finally, the sociocultural domain included: (i) language barriers, (ii) social exclusion (extreme poverty), (iii) belief conflict (spiritual distress), and (iv) lack of caregiver support.

We also collected information regarding the demographic and clinical characteristics of the patients, sex, underlying disease, continuity of care (discharged to another facility), length of hospital stay, and admission to ICU during hospitalization. Facilities were classified into two categories: high-tech hospitals or other. High-tech hospitals were defined as referral university centres that provide tertiary care for either open-heart surgery or major organ transplants.

### Statistical analysis

Descriptive analysis of data was performed to describe the patients’ demographic and clinical characteristics. For categorical variables, a comparative analysis for detecting significant differences between each age group was carried out using the Fisher’s exact test. For continuous variables the Mann-Whitney U test was used. Univariate multinomial logistic regressions of each CCIFs were performed to detect which ones were potentially associated with the different age groups (Table 2). The young-old (65–74) group was used as the reference category. The findings obtained were corroborated after adjusting for potential confounders: sex and hospital type (high-tech hospital) (Supplementary Material). The median-based CCIF categories shown in Table 2 were included solely for descriptive purposes and were not used in any regression models. The results of the logistic-regressions analyses were reported as odds ratios (OR) and 95% confidence intervals (CI). P values less than 0.05 were considered statistically significant. All reported p values are two-tailed. Statistical analysis was performed using the SPSS software package version 25.0 (SPSS, Chicago, IL).

## Results

A total of 5,658 older adult admissions were included. Of these, 46.3% (n = 2,622) were young-old (65–74 years), 34.8% (n = 1,971) middle-old (75–84 years) and 18.8% (n = 1,065) oldest-old (≥85 years) inpatients with COVID-19.

The baseline characteristics among patients hospitalized with COVID-19 according to the age category of older adults are presented in Table 1. Female sex and being discharged to another facility were more common in the oldest-old (≥85 years) than the other groups. Almost 91% of patients in the oldest-old group (≥85 years) presented underlying disease, mainly arterial hypertension or chronic heart failure (56.4%), diabetes or chronic kidney disease (47.9%) and chronic respiratory disease (19.2%) (p < 0.05). Conversely, length of stay (8 vs 9 days), admission to step-down unit (3.1% vs 12.6%) and transfer to ICU (6.3% vs 34.6%) were less common in the oldest-old (≥85 years) than in the young-old group (65–74 years) (p < 0.001).

**TABLE 1:** Baseline characteristics among patients hospitalized with COVID-19 according to the age category of older adults.

Characteristics	Study population n = 5,658		Young old (65–74) n = 2,622		Middle-old (75–84) n = 1,971		Oldest-old (≥85) n = 1,065		*p-value*
	No.	(%)	No.	(%)	No.	(%)	No.	(%)	
*Demographic characteristics*									
Age (years)_mean (SD)	76.5	(7.9)	69.5	(2.9)	79.0	(2.9)	88.9	(3.3)	**<0.001**
Female sex	2,414	(42.7)	1,042	(39.7)	809	(41.0)	563	(52.9)	**<0.001**
*Clinical characteristics*									
Length of stay_median (IQR)	9	(5–15)	9	(6–17)	9	(9–15)	8	(5–13)	**<0.001**
Continuity of care (discharged to another facility)	1,068	(18.9)	418	(15.9)	388	(19.7)	262	(24.6)	**<0.001**
High-tech hospital	3,785	(66.9)	1,756	(67.0)	1,313	(66.6)	716	(67.2)	0.937
Step-down unit	558	(9.9)	330	(12.6)	195	(9.9)	33	(3.1)	**<0.001**
ICU	1,371	(24.2)	907	(34.6)	397	(20.1)	67	(6.3)	**<0.001**
Underlying disease									
Arterial hypertension or chronic heart failure	3,074	(54.3)	1,356	(51.7)	1,117	(56.7)	601	(56.4)	**0.001**
Diabetes or chronic kidney disease	2,175	(38.4)	822	(31.4)	843	(42.8)	510	(47.9)	**<0.001**
Chronic respiratory disease	1,035	(18.3)	430	(16.4)	401	(20.3)	204	(19.2)	**0.002**
Cancer	542	(9.6)	237	(9.0)	224	(11.4)	81	(7.6)	**0.002**
Neurodegenerative disease	115	(2.0)	22	(0.8)	50	(2.5)	43	(4.0)	**<0.001**
Chronic liver disease	73	(1.3)	37	(1.4)	25	(1.3)	11	(1.0)	0.650
Immunosuppression	42	(0.7)	27	(1.0)	15	(0.8)	0	(0.0)	**0.004**
Dementia	70	(1.2)	6	(0.2)	20	(1.0)	44	(4.1)	**<0.001**

Abbreviations: SD, standard deviation; IQR, interquartile range; ICU, intensive care unit. Bold values indicate statistical significance (p<0.05).

Regarding CCIFs, we observed that lack of caregiver support, hemodynamic instability, chronic disease, transmissible infection, mental status impairments, and fear or anxiety were the most common CCIFs presented. The multinomial analysis showed that middle-old (75–84 years) patients were associated with chronic disease, position impairment, urinary or fecal incontinence, anatomical and functional disorders, vascular fragility, involuntary movements, fear or anxiety and mental status impairments. Extreme weight, communication disorders, aggressive behavior, agitation and perception reality disorders were additional factors associated with the oldest-old (≥85 years) inpatients with COVID-19. The median number of CCIFs was higher in the oldest-old than in the other age groups (four in young-old [65–74 years]; six in middle-old [75–84 years]; seven in oldest-old [≥85 years] [OR:2.9; 95%CI:2.8–3.1; p < 0.001]) (Table 2). Similar results we obtained after adjusting for potential confounders (Supplementary Material).

**TABLE 2:** Association of CCIFs among patients hospitalized with COVID-19 according to the age category of older adults.

Characteristics	Study population n = 5,658		Young-old (65–74) n = 2,622		Middle-old (75–84) n = 1,971					Oldest-old (≥85) n = 1,065				
	No.	(%)	No.	(%)	No.	(%)	OR	95% CI	*p-value*	No.	(%)	OR	95% CI	*p-value*
*Care complexity individual factors (CCIFs)*														
*Comorbidity/complications*														
Hemodynamic instability	5,074	(89.7)	2,328	(88.8)	1,779	(90.3)	1.17	0.97–1.42	0.109	967	(90.8)	1.25	0.98–1.58	0.073
Transmissible infection	4,023	(71.1)	1,850	(70.6)	1,412	(71.6)	1.05	0.93–1.20	0.424	761	(71.5)	1.04	0.89–1.22	0.586
Chronic disease	4,621	(81.7)	1,974	(75.3)	**1,679**	**(85.2)**	**1.89**	**1.62–2.20**	**<0.001**	**968**	**(90.9)**	**3.28**	**2.61–4.11**	**<0.001**
Uncontrolled pain	1,165	(20.6)	528	(20.1)	412	(20.9)	1.05	0.91–1.21	0.52	225	(21.1)	1.06	0.89–1.27	0.499
Extreme weight	526	(9.3)	228	(8.7)	172	(8.7)	1.00	0.82–1.23	0.97	**126**	**(11.8)**	**1.41**	**1.12–1.77**	**0.004**
Position impairment	807	(14.3)	200	(7.6)	**294**	**(14.9)**	**2.12**	**1.75–2.57**	**<0.001**	**313**	**(29.4)**	**5.04**	**4.15–6.13**	**<0.001**
Urinary or faecal incontinence	906	(16.0)	189	(7.2)	**348**	**(17.7)**	**2.76**	**2.29–3.33**	**<0.001**	**369**	**(34.6)**	**6.82**	**5.62–8.29**	**<0.001**
Anatomical and functional disorders	409	(7.2)	115	(4.4)	**162**	**(8.2)**	**1.95**	**1.53–2.50**	**<0.001**	**132**	**(12.4)**	**3.08**	**2.38–4.00**	**<0.001**
Communication disorders	185	(3.3)	61	(2.3)	56	(2.8)	1.23	0.85–1.77	0.274	**68**	**(6.4)**	**2.86**	**2.01–4.08**	**<0.001**
Vascular fragility	159	(2.8)	36	(1.4)	**64**	**(3.2)**	**2.41**	**1.60–3.64**	**<0.001**	**59**	**(5.5)**	**4.21**	**2.77–6.42**	**<0.001**
Immunosuppression	42	(0.7)	27	(1.0)	15	(0.8)	0.37	0.39–1.39	0.345	0	(0.0)			
Involuntary movements	46	(0.8)	12	(0.5)	**19**	**(1.0)**	**2.12**	**1.02–4.37**	**0.043**	**15**	**(1.4)**	**3.11**	**1.45–6.60**	**0.004**
High risk of haemorrhage	30	(0.5)	11	(0.4)	14	(0.7)	1.70	0.77–3.75	0.190	5	(0.5)	1.12	0.39–3.23	0.834
Oedema	17	(0.3)	7	(0.3)	5	(0.3)	0.95	0.30–3.00	0.930	5	(0.5)	1.76	0.56–5.56	0.334
Dehydration	6	(0.1)	0	(0.0)	3	(0.2)	-	-	-	3	(0.3)	-	-	-
*Psycho-emotional*														
Fear/anxiety	441	(7.8)	169	(6.4)	**169**	**(8.6)**	**1.36**	**1.09–1.70**	**0.006**	**103**	**(9.7)**	**1.55**	**1.20–2.01**	**0.001**
Impaired adaptation	385	(6.8)	192	(7.3)	139	(7.1)	0.96	0.77–1.20	0.73	**54**	**(5.1)**	**0.68**	**0.49–0.92**	**0.014**
Aggressive behaviour	28	(0.5)	6	(0.2)	10	(0.5)	2.22	0.81–6.13	0.122	**12**	**(1.1)**	**4.97**	**1.86–13.27**	**0.001**
*Mental-cognitive*														
Mental status impairments	1,985	(35.1)	491	(18.7)	**765**	**(38.8)**	**2.75**	**2.41–3.15**	**<0.001**	**729**	**(68.5)**	**9.42**	**8.01–11.07**	**<0.001**
Agitation	104	(1.8)	25	(1.0)	30	(1.5)	1.61	0.94–2.74	0.082	**49**	**(4.6)**	**5.01**	**3.01–8.15**	**<0.001**
Impaired cognitive functions	8	(0.1)	5	(0.2)	3	(0.2)	0.80	0.19–3.34	0.757	0	(0.0)	-	-	-
Perception of reality disorders	19	(0.3)	3	(0.1)	8	(0.4)	3.56	0.94–13.43	0.061	**8**	**(0.8)**	**6.61**	**1.75–24.95**	**0.005**
*Sociocultural*														
Lack of caregiver support	5,658	(100)	2,622	(100)	1,971	(100)	-	-	-	1,065	(100)	-	-	-
Belief conflict	4	(0.1)	4	(0.2)	0	(0.0)	-	-	-	0	(0.0)	-	-	-
Language barriers	79	(1.4)	58	(2.2)	**18**	**(0.9)**	**0.41**	**0.24–0.69**	**0.001**	**3**	**(0.3)**	**0.12**	**0.04–040**	**<0.001**
Illiterate	6	(0.1)	0	(0.0)	4	(0.2)	-	-	-	2	(0.2)	-	-	-
Social exclusion	1	(0.0)	1	(0.0)	0	(0.0)	-	-	-	0	(0.0)	-	-	-
CCIFs count, median (IQR)	5	(4–6)	4	(3–5)	**6**	**(5–7)**	**2.24**	**2.12–2.36**	**<0.001**	**7**	**(5–8)**	**2.96**	**2.79–3.15**	**<0.001**

Abbreviations: CCIFs, care complexity individual factors; IQR, interquartile range; OR, odds ratio; CI, confidence interval. The dependent variable of the multinomial logistic regression is the group that each individual belongs to. The young-old (65–74) group was used as the reference category. Bold values indicate statistical significance (p<0.05).

## Discussion

This study shows that the oldest groups of patients (≥75 years) admitted with COVID-19 had more CCIFs than the young-old group (65–74 years). Moreover, several CCIFs related to comorbidity/complications, psycho-emotional, mental-cognitive, and sociocultural domains were associated with the older patient groups.

The proportions of the subgroups of older adults described in this study are very similar to those found in the overall Spanish population (Instituto Nacional de Estadística. Instituto Nacional de Estadística INE, 2024). Furthermore, the number of CCIFs was higher in the study population compared with previous studies of admitted COVID-patients, being highest among the oldest-old patients (Seven CCIFs in patients ≥85 years old vs Four CCIFs in patients ≥18 years old) (Adamuz et al., 2021). Chronic disease, position impairment, urinary or fecal incontinence, anatomical and functional disorders, involuntary movements, vascular fragility, extreme weight, communication disorders, fear or anxiety, aggressive behavior, mental status impairments, agitation and perception reality disorders were the CCIFs associated with the middle (75–84 years), and oldest-old groups (≥85 years). As described in other studies, older and very old hospitalized individuals exhibit a higher prevalence of comorbidities and physical deterioration (Szklarzewska et al., 2023; Casas-Rojo et al., 2020) such as incontinence or position impairments. Moreover, the presence of cognitive impairment or dementia has been associated with an increase in care management complexity, due to difficulties in communication and adapting to the hospital environment (Veronese et al., 2022; Covino et al., 2020; Tondo et al., 2021). Additionally, social isolation and emotional stress, which were exacerbated during the pandemic, negatively affected the wellbeing of older patients, potentially complicating the recovery process and increasing the demand for psychosocial support (McNabney et al., 2022; Hwang et al., 2020). Finally, ICUs were often overwhelmed, and prioritization decisions for admission to these units generated ethical controversies (Hostiuc et al., 2021). In our study, the oldest-old group (≥85 years) was the least admitted to the ICU. Mortality, readmission and post-discharge functional status were not collected in this study, but we considered that this should be one of the future lines of research.

This research included a large sample size hospitalized with COVID-19 with the aim of identifying CCIFs associated with older adults. Notwithstanding, this study excluded patients directly admitted to and discharged from the ICU because CCIFs were not included in their electronic health records, and we relied on compliance in completing the healthcare history. The CCIFs included in this study, like other tools such as the Comprehensive Geriatric Assessment (CGA), provide a holistic patient assessment that involves biological, functional, psycho-affective, and social domains (Veronese et al., 2022). The CCIFs and CGA provide tools that allow healthcare staff to identify patients with a high risk of complications, and therefore prior identification of complexity factors can help to implement strategies to improve health outcomes and quality of life for older adults (Zurlo and Zuliani, 2018). The broader health assessment should be implemented in care models (McNabney et al., 2022) in order to help quantify the care burden and to estimate safe staffing for older patients. This would help establish age-friendly health systems and encourage geroscience (Inouye, 2021). Future studies should evaluate the incremental value of CCIFs relative to established tools—such as frailty indices and the CGA—since our analysis did not compare their respective discriminative performance; moreover, because CCIFs can be automatically extracted from routine nursing electronic health records, whereas frailty scales and CGA usually require times-consuming bedside assessments, they could therefore facilitate wider use and implementation compared with other tools.

### Limitation

It is important to point out certain limitations of this research:

- Data quality is supported by standard nursing documentation protocols; nevertheless, certain elements—particularly sociocultural factors—may have under-recorded, and no specific data-quality audits were conducted for this study, which we acknowledge as a limitation. Missingness for the four sociocultural CCIFs ranged from 0.1% to 3%, indicating possible under-recording, consistent with findings from previous studies in non-COVID populations (Adamuz et al., 2020).
- This analysis did not adjust for other potential confounders—including vaccination status, pandemic wave, baseline disease severity, and received treatments—which may have influenced the observed associations.
- Future longitudinal research should capture post-discharge functional end-points—such as objective mobility or activities-of-daily-living measures—to determine whether a high CCIF burden, particularly in patients with chronic conditions like arterial hypertension or ischemic heart disease, translates into poorer functional recovery after COVID-19 hospitalization (Borchev et al., 2024).

## Conclusion

The oldest groups of patients (≥75 years) admitted with COVID-19 had more CCIFs than the young-old group. Moreover, several CCIFs related to comorbidity/complications, psycho-emotional, mental-cognitive, and sociocultural domains were associated with the older patient groups. CCIFs should be included in patient assessment in order to identify care complexity individual needs in older hospitalized patients.

## Data Availability

The datasets presented in this article are not readily available because Datasets are property of the Catalan Institute of Health; therefore, any data sharing will require that institution’s prior approval. Data may be available from the corresponding author upon reasonable request and with permission from the Catalan Institute of Health. Requests to access the datasets should be directed to jadamuz@bellvitgehospital.cat.
