# Supplementary material for: Association between care complexity individual factors and older inpatients with COVID-19: a cross-sectional study
Source: Front Aging. 2025 Aug 5;6:1524849. doi: 10.3389/fragi.2025.1524849 (PMC12361132; doi:10.3389/fragi.2025.1524849)
Supplement: Supplementary file 1 [file DataSheet1.docx]

| **Online Supplement.** Association of CCIFs among patients hospitalized with COVID-19 according to the age category of older adults, adjusted by sex and hospital type (high-tech hospital). | | | | | | | | |
| --- | --- | --- | --- | --- | --- | --- | --- | --- |
| **Characteristics** | **Middle-old**  **(75-84)**  **n=1,971** | | | **Oldest-old**  **(≥85)**  **n=1,065** | | | |  |
|  | **OR** | **95% CI** | ***p-value*** | **OR** | **95% CI** | ***p-value*** |  |  |
| ***Care complexity individual factors (CCIFs)*** |  | | |  |  |  |  |  |
| ***Comorbidity/complications*** |  | | |  |  |  |  |  |
| Hemodynamic instability | 1.17 | 0.97-1.42 | 0.100 | **1.28** | **1.00-1.63** | **0.047** |  |  |
| Transmissible infection | 1.05 | 0.92-1.20 | 0.462 | 1.03 | 0.88-1.22 | 0.683 |  |  |
| Chronic disease | **1.91** | **1.64-2.11** | **<0.001** | **3.49** | **2.78-4.39** | **<0.001** |  |  |
| Uncontrolled pain | 1.04 | 0.90-1.21 | 0.57 | 1.01 | 0.85-1.20 | 0.918 |  |  |
| Extreme weight | 1.00 | 0.81-1.23 | 0.98 | **1.34** | **1.06-1.69** | **0.013** |  |  |
| Position impairment | **2.12** | **1.75-2.56** | **<0.001** | **4.92** | **4.04-5.98** | **<0.001** |  |  |
| Urinary or faecal incontinence | **2.76** | **2.29-3.33** | **<0.001** | **6.52** | **5.36-7.93** | **<0.001** |  |  |
| Anatomical and functional disorders | **1.95** | **1.52-2.49** | **<0.001** | **3.05** | **2.34-3.96** | **<0.001** |  |  |
| Communication disorders | 1.23 | 0.85-1.77 | 0.274 | **2.89** | **2.02-4.13** | **<0.001** |  |  |
| Vascular fragility | **2.42** | **1.60-3.66** | **<0.001** | **4.34** | **2.84-6.62** | **<0.001** |  |  |
| Immunosuppression | 0.35 | 0.39-1.39 | 0.347 | - | - | - |  |  |
| Involuntary movements | **2.12** | **1.03-4.39** | **0.041** | **3.28** | **1.52-7.06** | **0.002** |  |  |
| High risk of haemorrhage | 1.71 | 0.77-3.78 | 0.184 | 1.20 | 0.41-3.48 | 0.736 |  |  |
| Oedema | 0.95 | 0.30-3.01 | 0.938 | 1.79 | 0.56-5.70 | 0.323 |  |  |
| Dehydration | - | - | - | - | - | - |  |  |
| ***Psycho-emotional*** |  | | |  |  |  |  |  |
| Fear/anxiety | **1.36** | **1.09-1.69** | **0.007** | **1.52** | **1.18-1.97** | **0.001** |  |  |
| Impaired adaptation | 0.96 | 0.76-1.20 | 0.71 | **0.67** | **0.49-0.91** | **0.012** |  |  |
| Aggressive behaviour | 2.30 | 0.81-6.14 | 0.121 | **5.05** | **1.88-13.56** | **0.001** |  |  |
| ***Mental-cognitive*** |  | | |  |  |  |  |  |
| Mental status impairments | **2.76** | **2.42-3.16** | **<0.001** | **9.66** | **8.20-11.38** | **<0.001** |  |  |
| Agitation | 1.60 | 0.94-2.74 | 0.08 | **4.87** | **2.98-7.95** | **<0.001** |  |  |
| Impaired cognitive functions | 0.80 | 0.19-3.34 | 0.756 | - | - | - |  |  |
| Perception of reality disorders | 3.57 | 0.94-13.47 | 0.060 | **6.74** | **1.78-25.61** | **0.005** |  |  |
| ***Sociocultural*** |  | | |  |  |  |  |  |
| Lack of caregiver support | - | - | - | - | - | - |  |  |
| Belief conflict | - | - | - | - | - | - |  |  |
| Language barriers | **0.40** | **0.24-0.69** | **0.001** | **0.12** | **0.04-0.38** | **<0.001** |  |  |
| Illiterate | - | - | - | - | - | - |  |  |
| Social exclusion | - | - | - | - | - | - |  |  |
| CCIFs count, median (IQR) | **2.26** | **2.14-2.38** | **<0.001** | **2.97** | **2.79-3.16** | **<0.001** |  |  |
| \| Abbreviations: CCIFs, care complexity individual factors; IQR, interquartile range; OR, odds ratio; CI, confidence interval.  The dependent variable of the multinomial logistic regression is the group that each individual belongs to. The young-old (65–74) group was used as the reference category. \| \| --- \| | | | | | | | | |
